# Determinants of Senecavirus A Pathogenesis: From Viral Genome to ANTXR1, Immunity, and Programmed Cell Death

**DOI:** 10.3390/v18080922

**Published:** 2026-08-21

**Authors:** Xiaozhan Zhang, Siyu Wang, Ping Lu, Runfan Zeng, Guoyang Li, Changyao Li, Yiting Li, Xiuqing Li, Jinxing Song, Pandeng Zhao, Yunze Guo, Chuanzhou Bian, Decheng Yang, Xiaoyang Yan

**Affiliations:** 1 College of Veterinary Medicine, Henan University of Animal Husbandry and Economy, No. 6 Longzihu North Road, Zhengzhou 450046, China; zhangxz@hnuahe.edu.cn (X.Z.);; 2 Ministry of Education Key Laboratory for Animal Pathogens and Biosafety, Henan Agricultural University, Zhengzhou 450046, China; 3 College of Veterinary Medicine, Henan Agricultural University, Zhengzhou 450046, China; 4 State Key Laboratory for Animal Disease Control and Prevention, Harbin Veterinary Research Institute, Chinese Academy of Agricultural Sciences, Harbin 150069, China

**Keywords:** Senecavirus A, pathogenesis, viral factors, antiviral immunity, cell death processes

## Abstract

Senecavirus A (SVA), an emerging causative agent of porcine vesicular disease, belongs to the genus Senecavirus of the family *Picornaviridae*. The virus has been circulating in pig herds in the USA dating back to 1988, evolved into clinically significant pathogenic SVA variants causing a pandemic in the USA and Canada since 2014, and thereafter gradually spread to the Americas and Asia. To date, most studies have illustrated the infection dynamics, epidemiology, diagnostic methods, and vaccine development, yet the molecular pathogenesis of SVA remains incompletely characterized. As a novel picornavirus capable of establishing persistent subclinical infections, SVA has been detected in tissues such as tonsils and testicles for up to 156 days post-infection, posing a substantial challenge to swine health and production systems. In parallel, SVA has gained attention as an oncolytic virotherapy candidate for neuroendocrine tumors due to its tumor-selective tropism. Therefore, elucidating the mechanisms underlying SVA pathogenesis will not only support the development of effective countermeasures for swine but also inform the engineering of recombinant variants with enhanced therapeutic potential for human oncology. This review comprehensively summarizes the current knowledge on viral and host determinants of SVA pathogenesis, from the viral genome, evolution, and recombination to ANTXR1 host immunity, and programmed cell death, and presents future research directions, aiming to identify key knowledge gaps to guide future studies on SVA.

## 1. Introduction

Senecavirus A (SVA), a member of the *Senecavirus* genus within the *Picornaviridae* family, is a non-enveloped, positive-sense, and single-stranded RNA virus. Although being first incidentally identified as a contaminant in a culture of PER.C6-transformed retinoblastoma cells in 2002 [1], SVA was almost simultaneously confirmed to be responsible for vesicular lesions and epidemic transient neonatal losses in pigs in 2015 in USA and Brazil [2,3]. The clinical symptoms were characterized as acute lameness and coronary band vesicles in weaned and adult pigs, and lethargy, cutaneous hyperemia, diarrhea, neurological signs, and even sudden death in newborn piglets [2,3,4]. Thereafter, numerous swine-producing countries, including Canada, China, Colombia, Thailand, Mexico, Vietnam, England, Chile and India, have reported increasing outbreaks of Senecavirus-associated vesicular disease (SAVD) [5,6,7,8,9,10,11,12]. SVA has thus emerged as a globally prevalent virus, causing SAVD in pigs of all ages and sexes, and resulting in substantial economic losses to the swine industry [4,6,13].

**Figure 1:**
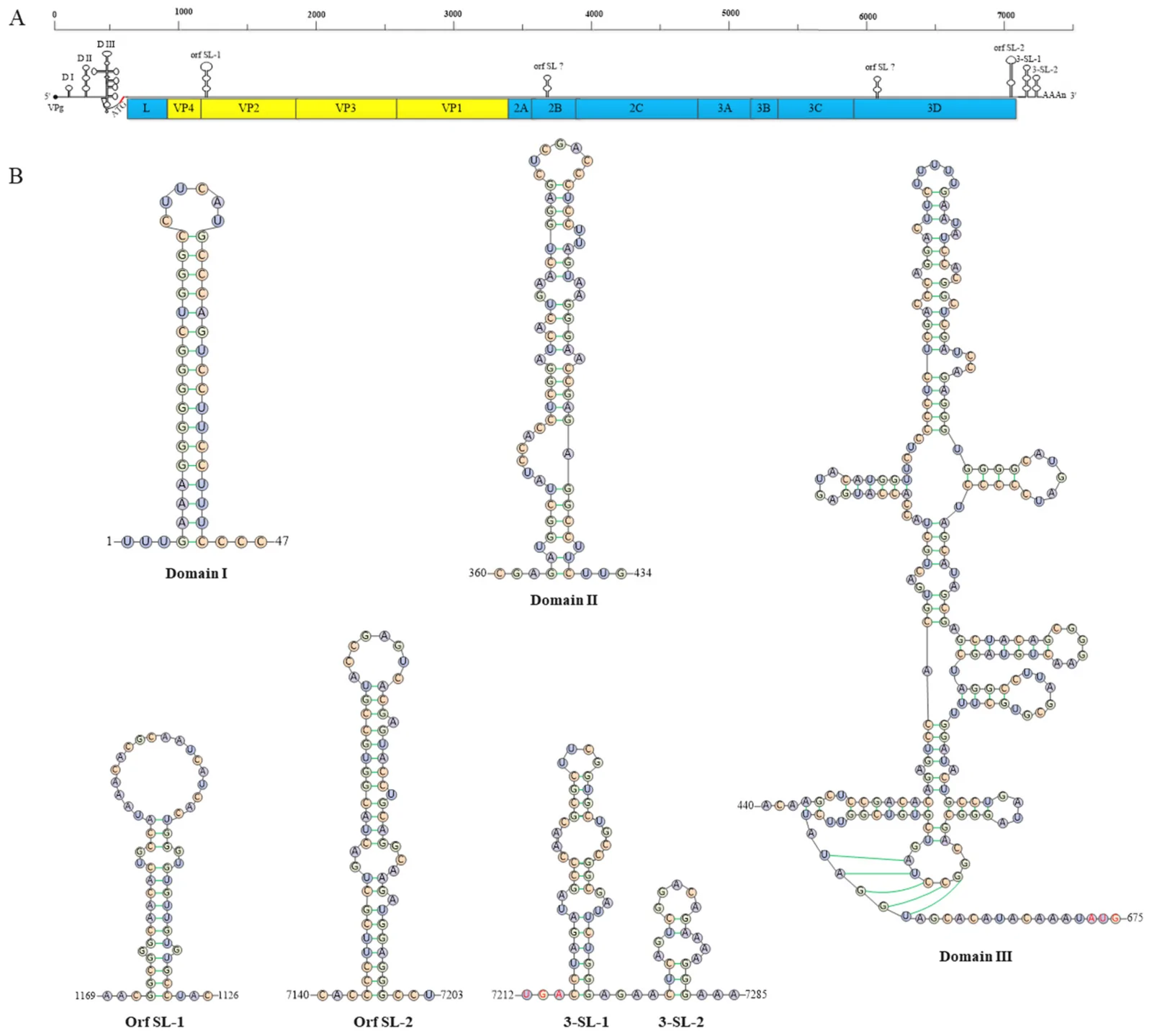
**Schematic representation of the SVA genome and its RNA secondary structures.** (**A**) Genomic organization of SVA. The positive-sense, single-stranded RNA genome is approximately 7.3 kb in length, featuring a 5′-terminal VPg protein and a 3′ poly(A) tail. Seven highly conserved stem-loop (SL) structures are highlighted, including three in the 5′ UTR, two within the ORF, and two in the 3′ UTR. (**B**) Functionally characterized SL structures. Depicted are the identified SL RNA secondary structures (Domain I, Domain II, Domain III, Orf SL-1, Orf SL-2, 3-SL-1 and 3-SL-2) that are critical for viral biological functions. The green line denotes an intramolecular covalent interaction that stabilizes the tertiary RNA architecture.

The SVA genome is approximately 7.3 kb in length and composed of a large single open reading frame (ORF) with a 3′ poly(A) tail but without a 5′ capped structure. The 5′-untranslated regions (5′-UTR) contains a sequence of the type IV internal ribosome entry site (IRES), which is similar to that of the classical swine fever virus (CSFV) and hepatitis C virus (HCV), and functions as assistance in the cap-independent initiation of protein synthesis [14]. The 3′-UTR is about 70 nt in length, with two stem-loop structures followed by a poly(A) sequence [15,16]. The ORF is translated into a single polyprotein, which is cleaved by virus-encoded protease into 12 protein products in a typical picornavirus L-4-3-4 layout (5′-L-VP4-VP2-VP3-VP1-2A-2B-2C-3A-3B-3C-3D-3′) [1] (Figure 1A). The structural proteins VP1, VP2, and VP3 jointly form the outer shell, which initiate infection via binding to specific cellular receptors such as anthrax toxin receptor 1 (ANTXR1). In contrast, VP4 is located on the inner surface of the capsid and plays a critical role in viral assembly [17].

Apart from the four structural proteins, the eight nonstructural proteins have been extensively characterized and found to be deeply involved in protein processing and viral replication [18]. Therein, L protein is the first protein encoded by SVA and possesses self-cleavage activity. The 2A protein is a short peptide of nine amino acids ending in an NPG/P conserved motif and performs the function of ribosomal skipping. Although the primary sequence of the 2B protein is dissimilar to that of other picornaviruses, its secondary structure is analogous to that of members in the same family, and likely plays a role in enhancing membrane permeability. The 2C protein functions as a helicase associating with RNA synthesis. The 3A protein is a small hydrophobic membrane protein that is involved in the formation of the viral replication complex. 3B encodes a viral protein (VPg) covalently linked to the 5′ end of the viral RNA genome and acts as a protein primer for RNA synthesis. 3C is a protease and responsible for polyprotein precursor cleavage. 3D is a component of RNA-dependent RNA polymerase (RdRp), and plays a critical role in virus replication and uridylation [18]. Owing to the sophisticated regulation of the viral genome and its encoded proteins, they may jointly contribute to the pathogenicity of SVA via both direct and indirect mechanisms.

In addition to being an animal pathogen, SVA has been selected as an oncolytic virus therapy against tumors with neuroendocrine features due to its oncolytic potential [19]. However, its safety and effectiveness still need to be further explored before successful clinical application. Accumulating data showed that SVA has been deeply involved in the swine industry and public health; therefore, elucidating its pathogenesis will undoubtedly facilitate the development of effective vaccines and antineoplastic methods to control the disease and benefit mankind.

In this review, we provide a comprehensive overview of the viral and host determinants of SVA virulence and pathogenesis, including viral factors (viral genome, evolution, and recombination) and host factors (viral receptors, host immunity, and programmed cell death, which influence viral replication). We further elaborate their involvement in antiviral immunity and programmed cell death, and present future research directions, aiming to integrate the current knowledge to inform future research on SVA.

## 2. Roles of Viral Factors in the Pathogenesis of SVA

### 2.1. Viral Genome RNA Structure Is Vital for the Replication of SVA

The SVA 5′-UTR is approximately 668 nt in length, and harbors an HCV-like IRES. With the help of covalent VPg at the 5′ end, the genome functions as an mRNA, and allows ribosomal subunits to be recruited to initiate protein expression in a cap- and eukaryotic initiation factor (eIF) 4E-independent manner [20]. The HCV and SVA IRES elements share most of the same RNA structure scaffold that contains two major hairpins, termed domain II (IIa and b) and III (IIIa, b, c, d, e, and f) [21,22]. Domain IIId includes two stem-loop structures, termed sub-domain IIId1 and 2, directing the interaction of IRES with the 40S small ribosomal subunit to initiate translation [23]. Domain IIIf is an H-type pseudoknot upstream of the start codon, formed by two base-paired stem regions (PKS-I and -II). PKS-I is composed of two base-paired motifs (PKS-Ia and -Ib), between which is a 2-base-unpaired spacing. PKS-II is a 5-base-paired stem structure, containing a non-Watson–Crick base pair (G:U base pair) to a stretch of complementary nucleotides elsewhere in the RNA chain [21] (Figure 1B).

Precise RNA sequence and secondary structure is vital for virus growth, and several studies have highlighted the key roles of IRES function in SVA replication. Mutant nucleotides were individually introduced into Domain II to Domain IIIf to change the stem-loop structures, and dual-luciferase reporter assay showed that, with the exception of Domain II, the mutations in the domains greatly inhibited the luciferase expression, suggesting that each subdomain in DIII had lower abilities of tolerating mutagenesis and was closely evolved in the IRES activity. Not surprisingly, no virus was successfully rescued from the eight mutant cDNA clones, implying that each of the putative domains was necessary in SVA IRES for viral replication [24]. Further detailed research showed that DIIId2 was dispensable for IRES activity but required for efficient SVA RNA synthesis and growth [25]. Similarly, motif mutations in PKS-I and PKS-II of DIIIf did not abolish the IRES function to initiate protein expression, but significantly interfered with SVA recovery from cDNA clones [26,27,28]. A 13 nt sequence between PKS-II and start codon AUG was highly conserved among wild-type SVA isolates. One-by-one single-nucleotide deletion in this 13 nt sequence within the SVA minigenome revealed that single-nucleotide deletion from 1 to 13 nt did not block IRES activity; only 1-nt-deleting SVA was successfully rescued and showed similar growth kinetics to that of wild-type SVA, with other sites’ mutation or deletion either failing to generate an infectious virus or producing viable progenies but with self-repaired nucleotide defects, suggesting that the sequence between the pseudoknot and AUG was crucial for SVA replication [15,29]. Although some conclusions are contradictory due to different strains or detection methods, all these data suggest that 5′-UTR plays critical roles in SVA recovery and replication. Further study should focus on the integrative effects of 5′-UTR in the SVA pathogenicity, and emphasize the synergistic function of different subdomains.

Apart from 5′-UTR, the 3′ terminal region of the SVA genome, located upstream of the poly(A) tail, is predicted to contain three conserved higher-order stem-loop structures. One of these is situated at the terminus of the 3D coding sequence (Orf SL-2), while the other two are located within the 68-nucleotide-long 3′-UTR (3-SL-1 and 3-SL-2). Different point mutations were individually introduced to evaluate their effects on virus recovery and replication. Results indicated that all three stem-loop structures were dispensable for SVA replication, as partial corresponding mutations did not prevent the recovery of an infectious virus. However, accumulated mutations that disrupt any of the stem-loop structures were found to abolish the recovery of an infectious virus [16,30,31]. Similarly, multiple conserved RNA structures that modulate viral replication and growth have been identified within the genomes of other picornaviruses, such as FMDV and EV71 [32,33]. These findings provide valuable insights for elucidating the cis-acting RNA elements required for SVA replication, which warrant further in-depth investigation. Taken together, these results demonstrate that the SVA genome harbors several cis-acting RNA elements essential for virus replication, particularly the putative stem-loop structures located within the 5′- and 3′-terminal regions.

### 2.2. Genetic Evolution and Recombination Alters the Infectious of SVA

In the past decades, SVA continued to evolve genetically. Genomic mutations, insertions, and recombination events have contributed to the emergence of SVA strains with divergent infectious profiles [5,13,34,35]. Furthermore, subclinical infections and viral carriage in healthy pig populations have been documented across multiple regions [5,35,36], contributing to the increasing complexity of SVA epidemiology. This trend is particularly evident in major swine-producing countries worldwide, including USA, China, Canada, and Brazil. The epidemiological and evolutionary dynamics of SVA in China provide a compelling example of this pattern. The first case of SAVD in China was reported in Guangdong province in 2015, presenting with vesicular lesions in sows and acute mortality in neonatal piglets [6]. Since then, SVA continuously spread to other provinces, such as Hubei, Fujian, Henan, and Guangxi [36,37], indicating its efficient infectious capability. Phylogenetic analysis demonstrated that all Chinese SVA strains clustered into two distinct lineages, comprising five genetic clades. The majority of Chinese strains grouped within clades I, II, III, and IV, which evolved closely with the USA-like lineage, while clade V was associated with the Canada-like lineage [13,36]. Although SVA strains from both the USA- and Canada-like lineages are capable of infecting pigs and inducing SAVD, differences in their pathogenicity have been observed. Data showed that USA-like lineage strain CH/AH-02/2017 and Canada-like lineage strain HB-CH-2016 displayed similar in vitro replication and plaque phenotypes, yet CH/AH-02/2017 induced significantly more severe clinical signs, and higher viremia and viral shedding in infected pigs, indicating that it displayed higher pathogenicity to pigs than HB-CH-2016 [37]. In addition, another study evaluated the pathogenicity of the classical USA-like lineage strain (US-15-41901SD), the classical Canada-like lineage strain (11-55910-3), and the ancestral strain (SVV-001), which revealed that US-15-41901SD induced markedly higher clinical scores, viremia, and viral shedding, and the cross-neutralizing antibody titers among the three infected groups differed markedly [38]. Genomic variations between these lineages, particularly amino acid substitutions in regions critical for ANTXR1 receptor-binding regions, were closely associated with the phenotypic divergence.

Since the first natural recombinant SVA strain, HeN-1/2018, was identified in China [39], the identification of recombinant SVA strains and the mechanisms underlying their formation have been extensively studied through both bioinformatic analyses and experimental approaches. In nature, recombination events have been identified across multiple geographical regions, including China, USA, Canada and Brazil, with breakpoints frequently localized to the P1 (capsid-encoding), 2C–3A junction, and 3C–3D junction regions (Table 1) [5,40,41,42,43,44,45]. Notably, the genomic breakpoints of several recombinant strains, including HeN-1/2018, seHN-3-2/2024, SVA/BRA/PR/446/22, SVA/BRA/PR/490/22, CH-GDSG-2018-3, 11-55910-3, and USA/HI13-007758/2013, were essentially mapped to the ANTXR1 receptor-binding domains, which contain the BC loop and loop II of VP1, the puff region of VP2, and the knob domain of VP3. Significantly, the seHN-3-2/2024 strain was derived from the USA-like and Canada-like lineages, and exhibits recombination-driven substitutions in the VP2-VP3-VP1 region within the breakpoints, including the key VP1 V93A substitution that reduces ANTXR1 receptor binding, which likely accounts for the observed attenuation of virulence [40,46]. Unfortunately, although numerous recombinant SVA strains have been identified, especially those harboring genomic changes in the ANTXR1 receptor-binding regions, the functional consequences of these recombination events on receptor binding, viral replication, and pathogenicity remain completely unexplored. This critical issue warrants sufficient attention in future study on SVA pathogenesis.

Recombination among SVA strains depends on the viral copy-choice recombination mechanism and the fidelity of the viral RdRp. Co-transfection of two different cDNA clones carrying lethal mutations in the IRES into BSR-T7/5 cells could generate wild-type SVA that reverted the IRES defects, providing evidence for the copy-choice recombination mechanism during SVA genome replication [24], thus ensuring the retention of replication-competent genomes. Recombination assays further demonstrated that SVA inter-genomic recombination is highly dependent on the fidelity of the viral RdRp, with the 3D S460L and I212V mutations enhancing fidelity and significantly reducing the recombination rate [47]. These findings may collectively provide clues that explain the origin and formation mechanisms of the subclinically circulating SVA strains currently prevalent in pig herds. However, it should be noted that the currently available evidence on SVA recombination is derived primarily from bioinformatic predictions, whereas functional validation of the phenotypic impact of recombinant strains remains a critical gap in the field.

## 3. ANTXR1 Mediates the Cellular Tropism and Pathogenesis of SVA

Anthrax toxin receptor 1 (ANTXR1), also known as tumor endothelial marker 8 (TEM8), serves as an essential cellular receptor for SVA infection [17]. ANTXR1 is a type I transmembrane protein with three domains, namely an extracellular N-terminal von Willebrand factor A (vWA) domain, a transmembrane domain, and a C-terminal cytoplasmic domain [48]. The vWA domain plays a critical role in mediating viral attachment and uncoating. Notably, two N-linked glycosylation sites within this domain, N166 and N184, were identified as essential posttranslational modifications for establishing stable interactions with SVA, since deglycosylation of ANTXR1 prevented virus attachment and subsequent entry [49]. The crystal structure of the SVA-ANTXR1 complex revealed that ANTXR1 decorates the outer surface of the viral capsid, engaging key structural motifs including the BC loop and loop II of VP1, the puff region of VP2, and the knob domain of VP3 [50]. Despite high structural and sequence similarity between ANTXR1 and its homolog ANTXR2, the capsid footprint on the receptor was not conserved in ANTRXR2, explaining the exquisite receptor selectivity of SVA for ANTXR1 [50].

Of note, the role of ANTXR1 as a critical host factor in initiating SVA infection has been demonstrated through a series of in vitro and in vivo experiments. In vitro, knockout of ANTXR1 gene in ST-R cells, a porcine cell line permissive for SVA replication, abolished SVA infection, whereas overexpression of ANTXR1 significantly enhanced SVA infection [51]. Knockdown of ANTXR1 expression in porcine alveolar macrophage cells significantly reduced viral infection and replication [52]. Moreover, knockout of ANTXR1 gene in non-porcine cells, including HAP1 and H449 cells, similarly abolished susceptibility to SVA, suggesting that ANTXR1 plays a crucial role in SVA infection. In vivo, CRISPR/Cas9-mediated knockout of the ANTXR1 gene in pigs completely blocked SVA infection in both the animals and their fibroblasts, and in pigs carrying in-frame mutations of ANTXR1, SVA infection was markedly reduced, with significantly lower clinical scores and viral shedding compared to wild-type pigs post-infection [53], further confirming that ANTXR1 is a key host factor mediating SVA infection.

Recently, an infectious SVA strain was isolated from buffaloes [54], raising concerns about the persistence of SVA in non-porcine animals and insects. Phylogenetic analyses revealed that the buffalo-derived SVA strain clusters closely with strains isolated from pigs, and animal challenge experiments confirmed that it can induce typical vesicular lesions in both piglets and buffaloes, indicating a potential for cross-species transmission [54,55]. Beyond buffaloes, SVA RNA or virus has been detected or isolated in mouse feces, small intestine, houseflies, and Culicoides midges from affected farms and even farms with no history of vesicular disease [56,57,58]. Although it remains unclear whether houseflies and Culicoides serve only as mechanical vectors, as evidence for active viral replication within these insects is limited, it is clear that mice, besides pigs and buffaloes, represent a third host species capable of supporting SVA replication and shedding, with SVA replication in mice significantly less efficient than in pigs [59]. However, whether this reduced susceptibility is due to the species-specific ANTXR1 gene divergence or other host factors remains unclear, as no comparative studies on ANTXR1 gene and protein structure among these species, or whether these differences affect the critical regions involved in SVA binding, have been reported. Further investigation is needed to clarify these unresolved issues.

## 4. The Interplay Between SVA and Host Immunity During Infection

### 4.1. The Host Antiviral Immune Response Restricts SVA Infection

**Figure 2:**
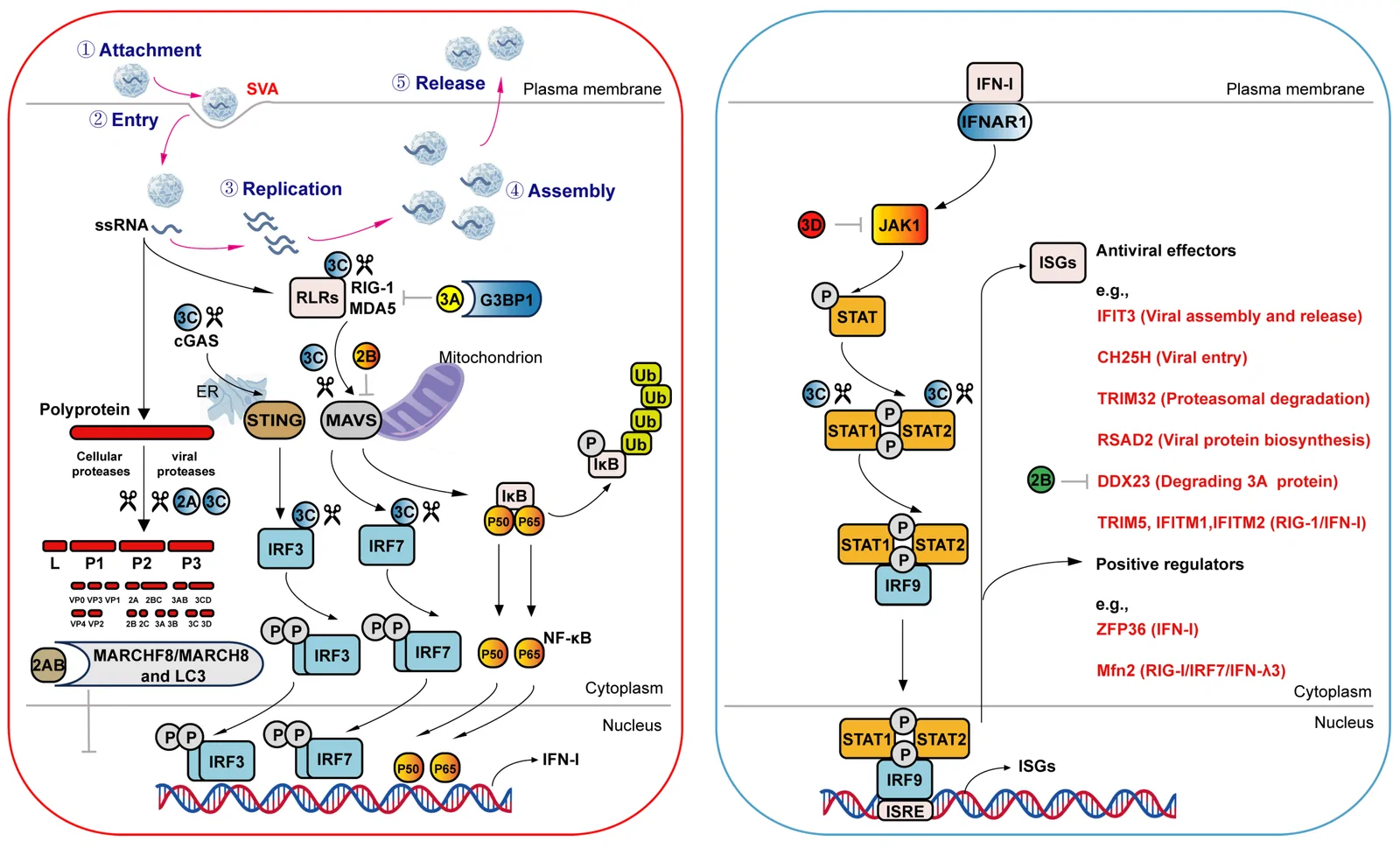
**Interplay between SVA and host antiviral immunity during infection.** Upon cellular entry, SVA genomic RNA is recognized by cytosolic RNA sensors including RIG-I and MDA-5, triggering activation of the type I interferon signaling pathway and subsequent JAK-STAT signaling cascade. This induction stimulates the expression of ISGs, such as IFIT1, IFIT3, CH25H, TRIM5, TRIM32, RSAD2, and ZFP36, which collectively restrict viral replication through multiple mechanisms. In response, the viral protease 3C and other viral proteins (2B, 3D, etc.) counteract host defense by targeting key immune adaptors, including RIG-I, MAVS, IRF3, IRF7, and STAT1, for degradation or functional inhibition, thereby attenuating the antiviral immune response.

Innate immunity acts as the very first line of defense for host cells against viral invasion and is an important prerequisite and basis for adaptive immunity to promote viral clearance [60]. Recent studies have demonstrated that type I and III interferon (IFN-I and IFN-III), mainly mediated by RIG-I-like receptors (RLRs), and the production of interferon-stimulated genes (ISGs) play important roles in host antiviral immunity and are necessary to block SVA infection [61,62]. Transcriptome profiles of PK-15 cells during SVA infection showed that RIG-I/MDA5 receptors initially recognized viral RNA, causing the up-regulation of IFN-III mediated by IRF7 [63]. Inhibiting IRF7 phosphorylation can reduce the production of IFN-λ3, but not IFN-λ1, and further promote SVA replication, suggesting that IFN-λ3 is more important in restricting SVA replication in vitro. Moreover, cellular protein Mfn2, a mitochondria-shaping protein, could inhibit the RIG-I/IRF7/IFN-λ3 signal pathway and facilitate SVA replicate in PK-15 cells [64]. Similarly, zinc-finger protein 36 (ZFP36) could promote SVA replication by suppressing the host IFN-I signaling pathway [65]. In contrast, the zinc-finger antiviral protein (ZAP) exerts multiple inhibitory effects on SVA. Its short isoform, ZAP-S, could interact with RIG-I to activate the downstream IFN-I signaling pathway, while the longer isoform, ZAP-L, could directly bind to the viral RdRp, leading to upregulated expression of IFN-β and IL-6 and consequently restricting SVA infection [66] (Figure 2).

As is well established, the host antiviral innate immune response largely depends on the coordinated functions of numerous ISGs. Proteome dynamics analysis showed that Mx1, an IFN-induced dynamin-like GTPase, was significantly upregulated in SVA infected PK-15 cells, and exerted antiviral activity through interacting with VP1, VP2, and VP3 proteins [67]. Apart from Mx1, IFIT3 acted as an anti-SVA factor downstream of IFN signaling via disrupting viral assembly and release [68]. Cholesterol-25-hydroxylase (CH25H) was shown to block SVA entry [69], while TRIM32 restricted SVA replication by mediating the ubiquitination and proteasomal degradation of the VP3 protein [70] and RSAD2 suppressed viral replication by interacting with the SVA 2C protein and interfering with viral protein biosynthesis [71]. Other ISGs, including TRIM5, IFITM1, and IFITM2, were found to positively enhance the RIG-I-mediated IFN-I antiviral response, thereby collectively inhibiting SVA replication [72,73] (Figure 2).

Since the demonstration of outstanding antiviral effects of IFN-related immune response, SVA showed discrepant virulence in different cell lines. Previous study has shown that SVA replicates more rapidly in IBRS-2 cells than in PK-15 cells, which can be attributed to aberrant signal transduction between TBK1 and IRF3 within the RIG-I pathway in IBRS-2 cells [74]. In cell lines with gene knockout of key immune molecules such as RIG-I, IRF3, or IRF7, SVA replication was obviously enhanced [61,75]. In vivo, infection of type I interferon receptor-deficient (C57BL/6J *^IFNR-/-^*) mice with SVA could cause more severe gross and histopathological lesions, as well as higher viremia levels, compared to SPF C57BL/6J wild-type mice, further confirming that aberrant signal transduction of the IFN-related immune response significantly reduces the resistance of mice to SVA infection [76]. Collectively, these findings highlight the critical role of host innate immunity, particularly the RIG-I driven IFN-related immune response, in restraining SVA propagation and influencing viral pathogenicity in cells and animals.

### 4.2. SVA Antagonizes the Antiviral Immune Response During Infection

A common viral strategy to antagonize the host antiviral immune response is through the proteolytic cleavage of immune-related proteins by virus-encoded proteases. The polyprotein encoded by SVA genome undergoes a series of processing events, mainly mediated by the protease 3C, to produce the mature viral particles. The crystal structure showed that SVA 3C adopted a typical chymotrypsin-like fold, and consisted of two domains connected via a long loop over the rear surface of the molecule. Protein substrates entered the binding groove of 3C via electrostatic adhesion, followed by exact cleavage at the specific site [77]. SVA 3C has been reported to target a wide range of host immune and inflammation-related proteins, through direct proteolytic cleavage or alternative post-translational modifications, including degradation and deubiquitination, thereby suppressing type I interferon responses and modulating inflammatory processes to facilitate viral replication (Table 2). These substrates include key components of the type I interferon pathway, such as cGAS, RIG-I, MAVS, TRIF, TANK, IRF3, IRF7, STAT1, and STAT2 [78,79,80,81,82,83], as well as proteins involved in antiviral immune regulation, including HDAC4, DCP1A, GSDMA, the IL-1β precursor, RNA helicase DHX30, and DDX21 [84,85,86,87,88,89,90]. Furthermore, several critical host transcription and translation related proteins, including poly(A)-binding protein cytoplasmic 1 (PABPC1), nucleolin (NCL), heterogeneous nuclear ribonucleoprotein K (hnRNP K), and hnRNP A1, were cleaved and degraded by SVA 3C to either suppress the expression of host immune-related proteins or generate viral beneficial fragments that attenuate the host antiviral immune response [91,92,93,94]. Collectively, the protease activity of SVA 3C plays a crucial role to suppress host antiviral responses and facilitate viral replication or immune evasion in vitro, acting via direct cleavage or indirect regulation of transcription factors (Figure 2).

Pathogenicity is synergistically regulated by different factors, and other viral proteins have been reported to jointly contribute to SVA virulence. The SVA 2B protein interacts with MAVS, resulting in degradation that inhibits IFN-I expression [95]. The SVA 2AB protein binds to MARCHF8/MARCH8 and LC3 to degrade the latter and inhibit selective autophagy and IFN-I production [96]. The SVA 2C protein can block the RIG-I-mediated IFN-β immune response through degradation of cellular RIG-I and potent inhibition of IRF3 phosphorylation [79], but the precise mechanism in which SVA 2C mediates RIG-I degradation remains unknown. The SVA 3A protein facilitates viral replication by degrading Ras-GAP SH3-binding protein 1 (G3BP1) through up-regulation of leucine-rich repeat-containing protein 25 (LRRC25), thereby suppressing RIG-I and MDA5 expression [97]. Independently, the SVA 3A protein serves as a target of the host antiviral protein DDX23, which undergoes degradation via the caspase-2/-6 pathway. Conversely, the 2B protein counteracts this degradation through the caspase-2/-3 pathway, ultimately suppressing DDX23-mediated antiviral defenses [98]. Furthermore, the 3D protein not only interacts with NLRP3, IKKα, and IKKβ to activate inflammasome assembly and IL-1β secretion, resulting in infected tissue hemorrhage and swelling [99], but also recruits the E3 ubiquitin ligase RNF125 to mediate K48-linked ubiquitination of JAK1, thereby negatively regulating JAK-STAT pathway activation [100] (Figure 2). Taken together, SVA can modulate host antiviral responses not only via the 3C protease but also through other viral proteins, including 2B, 2C, 3A, and 3D, collectively reprogramming the host antiviral immune response and establishing a cellular environment conducive to viral propagation.

### 4.3. The Host Adaptive Immune Response During SVA Infection

After SVA breaches the first line of innate immune defense and establishes infection in the host, the extent of the host subsequent adaptive immune response during infection becomes a critical determinant of viral pathogenicity and the infection outcome. Numerous factors influence the adaptive immune response, including immunization status, breed and age differences among infected animals, and the activation of T and B lymphocytes, etc. In vivo experimental infection of 6- to 10-day-old suckling piglets with SVA induced severe lymphocyte depletion in lymphoid tissues, including the spleen, tonsils, and mesenteric lymph nodes. This depletion was primarily attributed to the specific induction of B-cell apoptosis via the cleaved caspase-3 pathway, and accompanied by inhibition of B-cell proliferation, which collectively resulted in impaired humoral immune functions [101], and provided clues that explain why newborn piglets exhibit more severe clinical signs during SVA infection.

SVA infection can elicit robust humoral and cellular immune responses in pigs, with the magnitude of these responses varying among different pig breeds. Min and Landrace weaning piglets with SVV-CH-09-2018 revealed that Landrace piglets exhibited severe weight loss, 50% mortality and high levels of virus shedding in oral/nasal swabs, whereas Min piglets showed only mild weight loss, 10% mortality and low virus shedding. Notably, the IgA titer in the intestinal mucosa of infected Min piglets reached up to 200 doubling dilutions, which was significantly higher than that of infected Landrace piglets, along with increased M1 macrophage activation and elevated IFN-λ and IL-6 expression, indicating that Min pigs were more resistant to SVA infection, and highlighting breed-dependent differences in susceptibility and pathogenicity to SVA [102]. In non-immunized pigs, SVA induced early activation of T and B lymphocytes, leading to a robust virus neutralizing antibody (NA) response, which is strongly correlated with IgM responses against VP2 and VP3. and is accompanied by a marked increase in CD4^+^ T lymphocyte activation during the acute phase of infection. Importantly, the emergence of SVA-specific NA and CD4^+^ T lymphocyte activation coincided with the alleviation of clinical signs and decreased viral shedding in infected pigs [103]. These results indicate that SVA infection elicits robust adaptive immune responses in pigs, which are crucial for the control of SAVD, and the differential adaptive immune responses observed among pig breeds during SVA infection may contribute significantly to the difference in susceptibility to SVA.

Interestingly, T lymphocyte activation assays using PBMCs isolated from SVA-infected pigs demonstrated that the capsid proteins VP1, VP2 and VP3 each contain T-cell epitopes and contribute to SVA-specific cellular immune responses [103], providing a foundation for the dual evaluation of SVA-specific humoral and cellular immune responses of vaccinated animals in the efficacy assessment of candidate SVA vaccines. Both inactivated and VLP-based vaccines against SVA have been testified to induce sufficient neutralizing antibody (NA) titers and significant T lymphocyte activation in immunized pigs and mice [104,105,106]. In addition to the structural proteins VP1, VP2 and VP3, the nonstructural proteins 2C and 3A of SVA also contain specific T-cell epitopes (five in 2C and one in 3A). Co-immunization with inactivated vaccine elicited markedly enhanced epitope-specific IFN-γ^+^ T-cell responses and elevated NA titers at 28 days post-vaccination, indicating that these epitopes could boost the protective efficacy of the SVA-inactivated vaccine in pigs [107]. Collectively, these findings provide a scientific basis for SAVD control via SVA vaccination and offer valuable clues for developing novel synthetic peptide vaccines against SVA.

## 5. Reprogramming of Multiple Cell Death Processes Underlies SVA Pathogenesis

### 5.1. SVA Modulates Cell Autophagy Process

Autophagy is an evolutionarily conserved catabolic process that is critical to maintaining the stability of the intracellular environment [108]. Although numerous studies showed that autophagy plays key roles in antiviral immunity, members of different virus families, such as foot-and-mouth disease virus, influenza virus, and Peste des petits ruminants virus, have been reported to ignite autophagy to promote replication [96,109,110]. In PK-15 and BHK-21 cells, SVA induces autophagy via the PERK and ATF6 pathways, and negative regulation of SVA-induced autophagy response, achieved by treatment with 3-methyladenine (3-MA) or knockdown of endogenous ATG7 or LC3 expression, resulting in reduced SVA replication [111]. During SVA infection, SVA VP1, VP3 and 3C synergistically activate autophagy via the AKT-AMPK-MAPK-MTOR signal pathway to promote SVA replication [112]. However, the induction of autophagy inhibited SVA propagation in human 293T and H1299 cells, with SQSTM1 targeting VP1 and VP3 for autophagic degradation, which contrasts with its pro-viral role in porcine cells. SVA 3C counteracts this restriction by mediating cleavage of SQSTM1 at G355, G392 and G395, which abolishes its ability to mediate selective autophagy and thus restores SVA replication [113]. These findings indicate that the host exploits autophagy to modulate SVA infection, which exhibits species-dependent differences, and this variability may partially contribute to the variable pathogenicity of SVA across species. Nevertheless, the specific mechanisms require further confirmation through the generation of gene-edited animal models targeting autophagy-related factors and subsequent in vivo challenge experiments.

### 5.2. SVA Modulates Programmed Cell Apoptosis Process

SVA can regulate apoptosis, a programmed cell death process that is critical for maintaining cellular and host homeostasis, via both extrinsic and intrinsic pathways, both in vivo and in vitro. During SVA infection in piglets, a significant reduction in ki-67-positive cells was observed in the spleen, tonsils, and mesenteric lymph nodes, accompanied by enhanced caspase-3 immunoreactivity. These results indicate that SVA infection reduces lymphocyte counts in piglets through the induction of lymphoid apoptosis, with an apparent tropism for B lymphocytes. This mechanism may contribute to viral persistence in immune organs during prolonged infection [101]. In a separate mouse model of hepatocellular carcinoma (HCC), SVA potently suppressed tumor growth by inducing cell apoptosis and cell cycle arrest at the S phase, while exhibiting minimal cytotoxicity toward normal hepatocytes [114]. Meanwhile, the mechanisms by which SVA induces cell apoptosis have been elucidated in various cell lines in vitro studies. In 293T cells, SVA 2C and 3C play pivotal roles in virus-induced cell apoptosis. The mechanism involves 2C localizing to mitochondria and triggering caspase-3 activation to promote apoptotic signaling. Meanwhile, 3C depends on its protease activity to induce cell apoptosis by activating caspase-9, caspase-8, and caspase-3, ultimately leading to cytochrome C release into the cytoplasm, but not directly cleaving poly (ADP-ribose) polymerase family, member 1 (PARP1) [115]. In primary swine turbinate (STu) cells and human lung carcinoma (H1299), SVA 3C protein induces cell apoptosis by cleaving the transactivation domain (TAD) of NF-κB-p65 at 444L/Q445, thereby facilitating viral replication and/or release from infected cells during the late phase of SVA infection [116]. Notably, SVA infection induced endoplasmic reticulum (ER) stress in PK-15 and BHK-21 cells, triggering the translocation of Ca^2+^ from the ER to mitochondria. This cascade resulted in mitochondrial dysfunction and promoted mitochondria-mediated apoptosis, a process that ultimately enhances viral replication [117].

### 5.3. SVA Modulates Programmed Cell Pyroptosis Process

Besides cell apoptosis, cell pyroptosis, a form of inflammatory programmed cell death, is deeply involved in the antiviral immunity and pathogenesis of microbial infection. Previous studies have shown that several gasdermin (GSDM) family proteins, including GSDMB, GSDMC, GSDMD, and GSDME, can be cleaved by caspases or granzymes. Upon activation, these proteins form pores in the plasma membrane, a key step in the induction of cell pyroptosis [118]. The 3C proteins of picornaviruses have been demonstrated to modulate cell pyroptosis through cleavage of various gasdermin (GSDM) family proteins with distinct patterns [119]. In swine kidney (SK6) cells, SVA infection induces cleavage of porcine GSDMD and triggers cell pyroptosis in the presence or absence of caspase inhibitor Z-VAD-FMK, suggesting that SVA could elicit cell pyroptosis via both caspase-dependent and caspase-independent pathways. SVA 3C contributes to this process by cleaving NLRP3 to weaken inflammation while enhancing caspase-1 expression, and by directly cleaving GSDMD at Q193 and Q277 to generate a functional GSDMD_1–277_ fragment with similar cell-killing activity comparable to that of the canonical caspase-1 product. Interestingly, SVA 3C displays species specificity in GSDMD cleavage, being unable to cleave mouse or human GSDMD, with ectopic expression of porcine GSDMD switching the cell death manner from cell apoptosis to cell pyroptosis upon SVA 3C expression in 293T cells [87]. However, this species specificity does not extend to all GSDM family members. For GSDMA, SVA 3C can cleave both human and porcine GSDMA with comparable efficiency, producing a nonfunctional N-terminal fragment that counteracts the pore-forming activity of the full-length pyroptosis-inducing fragment, thus attenuating cell pyroptosis and lactate dehydrogenase release [86].

In summary, SVA infection exhibits species-dependent differences in both cell autophagy and cell pyroptosis, with special degradation mechanisms targeting regulatory factors involved in cell death processes in pigs, such as SQSTM1 and GSDMD. Moreover, SVA can selectively induce B lymphoid apoptosis in piglets. Collectively, these findings provide valuable clues for understanding the species- and age-dependent virulence manifestations during SVA infection. Beyond the cell death pathways discussed above, other programmed cell death processes, including necrosis, ferroptosis, and cuproptosis, may also be involved in SVA pathogenesis, although this remains speculative. The correlations among these events currently lack direct experimental evidence and warrant further investigation to elucidate their potential roles in SVA infection.

## 6. Conclusions and Future Prospects

Over the past decade, the novel emerging SVA has attracted extensive research attention due to its dual characteristics as an oncolytic virus capable of targeting tumor cells and as an infectious agent responsible for vesicular disease in pigs. Significant advances have been made in multiple areas, including molecular epidemiology, functional characterization of genomic RNA elements and viral proteins, viral reprogramming of cellular host processes, diagnostic and vaccine development, and mechanisms of oncolysis, collectively deepening our understanding of SVA pathogenesis. Recently, two inactivated vaccines against SVA were approved for commercial use in China, which is expected to substantially alleviate clinical control pressures. However, as a novel pathogen, many aspects of SVA virulence mechanisms remain incompletely understood. Deciphering this complexity will require novel mechanistic insights derived from cutting-edge systems biology approaches.

Regarding the aspect of genomic RNA function, the SVA RNA genome contains complex secondary structures, particularly within the 5′- and 3′-UTRs. Genomic mutations can modulate viral genome RNA secondary structures, with variable effects on SVA tropism and replication. In FMDV, another picornavirus that causes vesicular disease, a single-nucleotide substitution (C351G in the O/YS/CHA/05 strain) or 70 nt deletion (positions 148 to 217 in the O/BY/CHA/2010 strain) within the 5′-UTR altered the cell tropism and replication capacity of the parental strains, while significantly reducing their pathogenicity in host animals [120,121]. By analogy, mutations in the 5′-UTR region that impair SVA virulence may offer viable targets for live-attenuated vaccine development. It is noteworthy that internal genomic regions might also form intricate higher-order structures; however, the functional relevance of these structural elements in viral replication and pathogenicity remains largely unknown and warrants further investigation.

Regarding cellular host processes, as obligate intracellular parasites, viruses rely on host cellular machinery and manipulate multiple biological processes to establish a favorable intracellular environment for replication. It has been shown that SVA infection modulates host innate immune response, adaptive immune response, autophagy, cell apoptosis and cell pyroptosis; however, the effects of SVA on other critical cellular processes, such as necroptosis, ferroptosis, cuproptosis, energy metabolism, and key signaling pathways including PI3K/Akt, MAPK, and Wnt/β-catenin, remain poorly characterized and represent an important gap in the current knowledge.

Regarding viral surveillance and transmission, the global trade of live pigs and pork products constitutes a major component of agricultural commerce and poses a significant risk for the spread of SVA. Previous studies have confirmed that acutely infected boars shed SVA in semen, with the virus being detectable in routine semen screenings [40,122]. How SVA breaches the blood–testis barrier, whether the virus in semen remains infectious, and whether it can be transmitted via semen during artificial insemination are issues that remain unresolved. These issues represent critical unknowns that demand further experimental investigation.

## Figures and Tables

**Table 1:** Summary of global recombinant SVA strains of their strain details and recombination events.

Strain	Year	Location of Detection	Proposed Parental Lineages	Genomic Breakpoints	Detection Methods	References
HeN-1/2018	2018	China	USA/IA44952/2015 (Major) USA/IN_Purdue_4885/2015 (Minor)	VP4 (partial)-VP2-VP3 (partial)	RDP4, SimPlot	[39]
seHN-3-2/2024	2024	China	USA/IA09-34037/2009 (Major) Canada/MB/NCFAD-104-9/2015 (Minor)	VP2 (partial)-VP3-VP1-2A-2B (partial)	RDP4, SimPlot	[40]
SVA/BRA/PR/446/22	2022	Brazil	SVA/BRA/GO/236/21 (Major) SVA/BRA/PR/458/22 (Minor)	VP2 (partial)-VP3-VP1 (partial)	RDP5, SimPlot	[41]
SVA/BRA/PR/363/21	2021	Brazil	SVA/BRA/PR/384/22 (Major) SVA/BRA/PR/362/21 (Minor)	2B(partial)-2C-3A-3B-3C	RDP5, SimPlot	[41]
SVA/BRA/MT/11/19	2019	Brazil	SVA_Brazil_949_22 (Major) SVA/BRA/MT/39/19 (Minor)	VP2 (partial)-VP3-VP1 (partial)	RDP5, SimPlot	[41]
SVA/BRA/PR/595/22	2022	Brazil	SVA/BRA/GO/462/22 (Major) SVA/BRA/PR/587/22 (Minor)	2B(partial)-2C-3A-3B-3C (partial)	RDP5, SimPlot	[41]
SVA/BRA/PR/490/22	2022	Brazil	SVA/BRA/PR/489/22 (Major) SVA/BRA/PR/431/22 (Minor)	VP2 (partial)-VP3-VP1 (partial)	RDP5, SimPlot	[41]
SVA-CH-SDGT-2017	2017	China	USA/IA44952/2015-P1 (Major) CH-GDLZ01-2017 (Minor)	VP2 (partial)-VP3(partial)	RDP4	[42]
CH-GDSG-2018-3	2018	China	CHhb2017 (Major) HeNKF-1 (Minor)	VP2 (partial)-VP3(partial) VP3 (partial)-VP1(partial)	SimPlot	[43]
SVA/Canada/ON/FMA-028-9F/2016	2016	Canada	SVA/Canada/ON/FMA-025-2C/2016 (Major) SVA/Canada/ON/FMA-029-2D/2016 (Minor)	VP1(partial)-2A-2B-2C-3A-3B-3C-3D (partial)	RDP5	[5]
11-55910-3	2011	Canada	USA/MN99-29256/1999 (Major) SVA/Canada/MB/NCFAD-104-6/2015 (Minor)	5′UTR(partial)-VP4-VP2-VP3-VP1(partial)	RDP5	[5]
USA/HI13-007758/2013	2013	USA	USA/IA09-34037/2009 (Major) SVA/Canada/MB/NCFAD-104-9/2015 (Minor)	VP2(partial)-VP3-VP1-2A-2B (partial)	RDP5	[5]
USA/IL01-84124/2001	2001	USA	USA/LA97-98061/1997 (Major) USA/TN06-00310/2006 (Minor)	VP1(partial)-2A-2B-2C (partial)	RDP5	[5]
SVA/CHN/10/2017	2017	China	SVA/CHN/11/2017 (Major) SVA/CHN/01/2017 (Minor)	2C(partial)-3A-3B-3C (partial)	SplitsTree, RDP4, RDP5	[5,44,45]
CH-GDJY-2018	2018	China	SVA/CHN/07/2017 (Major) GD-ZYY02-2018 (Minor)	2C(partial)-3A-3B-3C-3D (partial)	SplitsTree, SimPlot, RDP4, RDP5	[5,44]
CH-GD-2017-2	2017	China	CH-GD-2017-1 (Major) CH-HN-2017 (Minor)	VP1(partial)-2A-2B-2C (partial)	SplitsTree, RDP4, RDP5	[5,44]
HeNNY-1/2018	2018	China	AH02-CH-2017 (Major) HeNZMD-1/2018 (Minor)	2C(partial)-3A-3B-3C (partial)	RDP4, SimPlot	[45]

**Table 2:** Post-translational modification of cellular host proteins by SVA 3C.

Host Cell Protein	Cleavage Site	Activity	Effect	References
cGAS	GAWK_138_LQTV (porcine)	Direct cleavage	Immune evasion	[78]
RIG-I	-	Degrade via the caspase pathway/Deubiquitinate of K63-linked polyubiquitin	Immune evasion	[79,80]
TBK1	-	Deubiquitinate of K48-linked polyubiquitin	Immune evasion	[80]
TRAF3	-	Deubiquitinate of K63-linked polyubiquitin	Immune evasion	[80]
MAVS	VQETQ_148_APESPG	Direct cleavage	Immune evasion	[81]
TRIF	IRTLQ_159_SNLGCL	Direct cleavage	Immune evasion	[81]
TANK	ME_272_FRDNPGNFVKTEETLFEIQ_291_G	Direct cleavage	Immune evasion	[81]
IRF3 and IRF7	-	Degradation by its protease activity	Immune evasion	[82]
STAT1	PMEL_693_D_694_GPKG	Direct cleavage	Immune evasion	[83]
STAT2	DELQ_707_QPL/LESV_754_L_755_E_756_S_757_TLE (human)/PMLQ_758_STL (porcine)	Direct cleavage	Immune evasion	[83]
HDAC4	LLEQ_599_QRIH	Direct cleavage/degrade via the caspase pathway	Immune evasion/promote replication	[84]
DCP1A	MMQ_343_AVKT	Direct cleavage	Promote replication	[85]
GSDMA	GLQG_187_S_188_INHKE	Direct cleavage	Promote replication	[86]
GSDMD	LQ_193_GQ……FQ_277_SD (porcine)	Direct cleavage	Promote replication	[87]
pro-IL-1β	ECK_123_L_124_QDK (porcine)	Direct cleavage	Promote inflammatory response	[88]
DHX30	Q_220_ (porcine)	Direct cleavage	Promote replication	[89]
DDX21	-	Degrade via the caspase pathway	Promote replication	[90]
PABPC1	Q_437_	Direct cleavage	Promote replication	[91]
nucleolin	Q_545_	Direct cleavage	Promote replication	[92]
HnRNP K	YEPQ_364_GGSG	Direct cleavage	Promote replication	[93]
HnRNP A1	-	Degrade via the proteasome pathway	Promote replication	[94]

## Data Availability

No new data are included in this article. Data in referenced prior studies may be accessible in the original works.

## Abbreviations

SVA	Senecavirus A
SAVD	Senecavirus-associated vesicular disease
ORF	open reading frame
UTR	untranslated regions
IRES	internal ribosome entry site
ANTXR1	anthrax toxin receptor 1
VPg	viral protein covalently linked to the 5′ end of viral RNA genome
eIF	eukaryotic initiation factor
SL	stem-loop structure
IFN	Interferon
RLRs	RIG-I-like receptors
ISGs	Interferon-stimulated genes
ZAP	zinc-finger antiviral protein
CH25H	cholesterol-25-hydroxylase
hnRNP	heterogeneous nuclear ribonucleoprotein
HCC	hepatocellular carcinoma
Stu	swine turbinate
ER	endoplasmic reticulum
GSDM	Gasdermin
vWA	von Willebrand factor A
CavME	caveolae-mediated endocytosis
